# Attitudes of Policy Makers in Hawaii toward Public Health and Related Issues before and after an Economic Recession in the United States

**DOI:** 10.3389/fpubh.2015.00146

**Published:** 2015-05-26

**Authors:** Jay E. Maddock, Meghan McGurk, Thomas Lee

**Affiliations:** ^1^Department of Public Health Sciences, University of Hawaii at Manoa, Honolulu, HI, USA; ^2^School of Public Health, Texas A&M University, College Station, TX, USA

**Keywords:** health policy, economics, decision makers, public health, Hawaii

## Abstract

**Introduction:**

Legislation and regulation at the state and local level can often have a greater impact on the public’s health than individual-based approaches. Elected and appointed officials have an essential role in protecting and improving public health. Despite this important role, little systematic research has been done to assess the relative importance of public health issues compared to other policy issues in times of economic hardship. This study assessed attitudes of elected and appointed decision makers in Hawaii in 2007 and 2013 to determine if priorities differed before and after the economic recession.

**Methods:**

Elected and appointed state and county officials were mailed surveys at both time points. Respondents rated the importance of 23 specified problems, of which 9 asked about specific public health issues.

**Results:**

The survey was completed by 126 (70.4%) respondents in 2007 and 117 (60.9%) in 2013. Among the public health issues, five saw significant mean decreases. These variables included climate change, pedestrian safety, government response to natural disasters, access to healthcare, and pandemic influenza. Obesity was the only public health issue to increase in importance across the two time points. In terms of relative ranking across the time points, only drug abuse and obesity were among the top 10 priorities. Lack of public health training, pandemic influenza, and government response to natural disasters were among the bottom five priorities.

**Conclusion:**

After the economic recession, many public health issues have a lower priority among Hawaii’s policy makers than before the downturn. Additional education and advocacy is needed to keep public health issues on the minds of decision makers during tough economic times.

## Introduction

Over the past century, there have been several major public health policy successes that have resulted in millions of lives being saved worldwide, including universal salt iodization, motorcycle helmet requirements, and smoke-free laws (1–3). As codified in the Ottawa Charter in 1986, there has been a clear charge to public health practitioners to advocate for health policies (4). However, research in the area has been limited, with <10% of articles published in leading health promotion journals focused on health policy issues (5).

A recent study estimated that there are over 80,000 preventable deaths in the United States each year (6). However, death rates varied dramatically by state indicating that differences in the policies, culture, health care, demographics, and environment in these states may contribute to excess deaths (6). The social-ecological model postulates that behavior is influenced by a variety of factors including interpersonal, intrapersonal, organizational, community, and public policy (7). The model is often depicted as a series of concentric circles with the outermost circle being public policy or the policy environment (7). The model proposes that policy has the potential to reach the highest number of people in the population of interest and is an essential part of multicomponent interventions to change behavior and reduce premature morbidity and mortality (7).

Public health practitioners and advocates need to understand the policy process and what influences the success or failure of certain bills. Kingdon has developed a three-stream model for examining the policy change process that includes politics, proposals, and problems (8).

Public health advocates are often working to influence health policy adoption. Politics, which involves the election or appointment of officials, is typically outside of the control of these groups but can be influenced, to some extent, by the election process. Proposals are specific bills or solutions to a problem. These are essential when the policymakers have identified a particular issue in need of change. However, one of the main concerns for health advocates and coalitions is how do public health issues become recognized as problems and put on the agenda for change. Agenda setting requires that issues be perceived as problems that need public action to address them (9, 10). Assessment of what policymakers think is important as well as what external factors influence public policy making is essential in understanding the success of advocacy efforts.

Public health advocacy efforts are essential at the community level. State and local coalitions are often unaware of how their efforts are influencing change. Identification of public health issues as a problem is usually needed before solutions and policies can be presented. Surveillance of attitudes of policymakers across time can provide important intermediate outcome data to public health advocates about the effects of their work.

In Hawaii, we were interested in the effects of advocacy efforts on attitudes toward public health issues in the state. In 2007, we conducted a baseline survey to assess the attitudes of elected and appointed officials at the state and county level (11). Our results showed that drug abuse, climate change, and pedestrian safety were rated as high priorities, while pandemic influenza, access to healthy groceries, and poor nutrition were not seen as priorities (11). Between 2007 and 2013, many changes took place within the state. Hawaii elected its first Republican governor since statehood in 2004. In 2012, a Democratic governor was elected. The governor is responsible for appointing all of the directors and deputy directors of state departments, so a major change in governance of the state occurred. In 2007, state and county physical activity and nutrition coalitions were founded with a mission focused on policy and environmental change (12). The Fukushima Daiichi tsunami and the H1N1 pandemic both occurred in the time frame. The “Great Recession” occurred in the United States between December 2007 and June 2009 with long-term effects including high unemployment rates continuing into 2012 (13). Hawaii, with its tourism dependent economy faced severe economic hardships including a $1.2 billion shortfall in fiscal year 2010 equivalent to over 25% of the total state budget (14).

Given the change in political party of the governor, two major global public health events and multiple public health advocacy efforts in the interim, we hypothesized an increase in importance for public health issues relative to other concerns, despite the Great Recession. The goal of this study was to assess changes in legislative priorities among elected and appointed officials in Hawaii between 2007 and 2013 to better inform future advocacy work.

## Materials and Methods

### Sample

Policymakers in Hawaii were surveyed in 2007 and 2013 to assess their attitudes toward priority issues for the State of Hawaii. Due to the state’s small size, a census sampling approach was used to select all Hawaii state and county elected officials as well as gubernatorial appointed officials of state departments and agencies. Appointed state-level officials included the directors and deputy directors of all state departments, and appointed board members of state agencies. State department heads included those of the Department of Health, Department of Transportation, Division of Land and Natural Resources, Department of Education, and the Office of Hawaiian Affairs, among others.

This led to a 2007 population of 185 positions, with 25 state senators, 51 state representatives, 2 executive branch members, 34 county council members, 4 mayors, and 69 appointed state-level officials. In 2013, the sample increased to 192 with the addition of 7 state department officials. All other position numbers remained the same.

All potential participants were mailed a survey along with a cover letter assuring confidentiality and a postage-paid return envelope. Two weeks later, non-responding participants with public emails were emailed a copy of the survey and cover letter, while those without public emails were mailed a second survey. After 1 month, follow up calls were made to the remaining non-respondents.

### Ethics

All study procedures were reviewed and approved by the University of Hawaii Committee on Human Studies.

### Survey

The survey used in this study was adapted from a previously published tool (15). Modifications were made to the instrument to include a range of current national and Hawaii-based public health and social welfare concerns. The surveys asked respondents to rate how much of a problem the 23 issues were in Hawaii (from 1 – not a problem to 5 – a problem of extreme importance). The 23 problems included 10 public health issues (climate change, drug abuse, access to health care, poor nutrition, access to healthy groceries, obesity, pedestrian safety, government response to natural disasters, lack of public health training, and pandemic influenza), six economic issues (lack of good jobs, poverty, lack of affordable housing, high taxes, homelessness, and cost of living) and seven infrastructure and general issues (ethics in government, quality of public education, poorly planned development and sprawl, lack of recreational activities, increasing traffic, lack of pedestrian walkways, crosswalks and sidewalks, and crime). Additional information on the 2007 instrument has been published elsewhere (11).

### Data analysis

The 23 close-ended questions were coded from −2 to +2 to aid interpretation. Two-tailed tests and an alpha level of <0.05 were used for all analyses to test significance. Rank orders by mean score were used to compare the relative importance across the two time points. Next, ANOVA and *t*-tests were used to assess differences in priorities by type of position and political party affiliation and to assess changes between the two time points. Then, we used bivariate correlations assess the how strongly correlated the ratings on the 10 public health issues were with how liberal or conservative individuals rated themselves on both fiscal and social issues (5-point scale; −2 = Liberal and +2 = Conservative). Cohort analyses were conducted using paired sample *t*-tests.

## Results

### Participants

After mailing out the 2007 survey, project staff was notified of six vacant appointed state department positions leaving a population of 179. Of these, 126 (70.4%) returned completed surveys. The respondents were 1 state executive, 15 senators, 34 representatives, 32 county officials, 43 appointed officials, and 1 unidentifiable participant. Among respondents with an official political affiliation, 46 were Democrats and 12 were Republicans.

After the 2013 survey distribution, two positions were removed from the sample due to vacancies. The resulting population included 192 positions, of which 117 (60.9%) returned completed surveys. The respondents were 1 state executive, 15 senators, 26 representatives, 21 county officials, 51 state-level officials, and 2 unidentifiable participants. Among respondents with an official political affiliation, 39 were Democrats and 3 were Republicans.

The unidentifiable participants were removed for further analysis leaving sample sizes of 125 and 115. There were no significant differences between the time points on position type, gender, or party affiliation. Table 1 summarizes the participant demographics.

**Table 1:** **Sample demographics**.

Category	2007 *n* = 125 *n* (%)	2013 *n* = 115 *n* (%)
**POSITION TYPE χ^2^(5) = 3.79, *p* = 0.43**		
Executive	1 (0.8)	1 (0.9)
Senate	15 (12)	15 (13.0)
House	34 (27.2)	26 (22.6)
State department	43 (34.4)	52 (45.2)
Mayors and county councils	32 (25.6)	21 (18.3)
**POLITICAL PARTY χ^2^(2) = 5.87, *p* = 0.054**		
Democrat	46 (36.8)	39 (33.9)
Republican	12 (9.6%)	3 (2.6%)
Non-partisan	67 (53.6%)	73 (63.5%)
**GENDER χ^2^(1) = 0.044, *p* = 0.83**		
Male	76 (61.3)	72 (62.1)
Female	48 (38.7)	43 (37.9)

Among the respondents, 33 people who completed the survey at both time points. A cohort dataset was developed to assess within person changes in this group.

### Cross-sectional results

Between 2007 and 2013, 5 of the 10 public issues significantly decreased in the mean rating for importance. This included drug abuse, access to health care, pedestrian safety, government response to natural disasters, and pandemic influenza. Only two issues significantly increased in importance, obesity and access to healthy groceries. Among the economic issues, only two of the issues changed significantly with concern about lack of good jobs increasing and lack of affordable housing decreasing. Among the infrastructure and general issues, a decrease in concern for poorly planned development and sprawl was the only significant change. Table 2 displays these results.

**Table 2:** **Cross-sectional importance of items between 2007 and 2013**.

Item	2007 m (sd)	2013 m (sd)	*p*-value	Rank 2007	Rank 2013
**PUBLIC HEALTH ISSUES**					
Climate change	0.95 (1.09)	0.72 (1.06)	0.087	8	10
Drug abuse	1.46 (0.69)	1.26 (0.73)	0.039	2	2
Access to healthcare	0.96 (1.02)	0.61 (1.05)	0.009	8	13
Access to healthy groceries	−0.44 (1.09)	−0.11 (1.02)	0.018	23	21
Pedestrian safety	0.82 (1.04)	0.30 (0.98)	<0.001	12	17
Lack of public health training	0.24 (0.86)	0.07 (0.85)	0.125	19	19
Government response to natural disasters	0.30 (1.02)	−0.13 (1.05)	0.001	17	22
Pandemic influenza	0.21 (0.90)	−0.07 (0.89)	0.018	21	20
Poor nutrition	0.22 (0.87)	0.30 (0.84)	0.445	20	16
Obesity	0.58 (0.83)	0.88 (0.88)	0.009	14	9
**ECONOMIC ISSUES**					
Lack of good jobs	0.71 (0.93)	1.20 (0.72)	<0.001	13	6
Poverty	0.90 (0.86)	0.98 (0.81)	0.493	10	8
Lack of affordable housing	1.63 (0.60)	1.37 (0.73)	0.003	1	1
High taxes	0.57 (1.03)	0.41 (1.08)	0.253	15	14
Homelessness	1.33 (0.65)	1.24 (0.73)	0.307	3	5
Cost of living	1.15 (0.88)	1.27 (0.86)	0.330	6	3
**INFRASTRUCTURE/MISCELLANEOUS**					
Ethics in government	0.29 (1.05)	0.22 (1.05)	0.593	18	18
Quality of public education	1.33 (0.80)	1.25 (0.76)	0.445	4	4
Poorly planned development and sprawl	1.00 (0.98)	0.70 (0.99)	0.022	7	11
Lack of recreational activities	−0.35 (1.13)	−0.30 (1.06)	0.733	22	23
Increasing traffic	1.26 (0.79)	1.05 (0.87)	0.052	5	7
Lack of pedestrian walkways, crosswalks and sidewalks	0.31 (0.95)	0.32 (0.97)	0.935	16	15
Crime	0.88 (0.89)	0.68 (0.85)	0.091	11	12

Among the public health issues, access to healthcare, pedestrian safety, and government response to natural disaster all dropped by five places in rank order. Only obesity (14–9) increased by more than two places. Among the 23 issues, 4 of the 5 bottom ranked issues in 2013 were related to public health (i.e., government response to natural disasters, access to healthy groceries, pandemic influenza, and lack of public health training).

Among the economic issues, lack of good jobs increased from 13th to 6th place. Lack of affordable housing was ranked first at both time points. Infrastructure and general issues remained generally the same except for poorly planned development and sprawl which dropped from 7 to 11. These results are displayed in Table 2.

Among the 10 public health variables, only pedestrian safety was significantly different by position (*p* < 0.05) with county officials (*m* = 0.65, sd = 0.88) rating it as more important than state-level appointed (*m* = 0.06, sd = 1.07) and elected officials (*m* = 0.43, sd = 0.84). The only significant difference was on access to healthcare (*r* = −0.23 social; −0.24 fiscal). We found the same results for beliefs on how often the government should restrict people’s behavior to protect health (*r* = −0.23 for access to health care) and increase taxes to protect health (*r* = −0.23).

### Cohort results

Only 11 of the 23 variables were significantly correlated between the time points (*r* ranged from 0.38 to 0.79). The only significant changes were decreases in the importance of drug abuse and government response to natural disasters, and an increase in concern for lack of good jobs.

## Discussion

This study examined changes in attitudes toward public health issues in 2007 and 2013. At both time points, public health issues ranked near the bottom of concerns among these officials. Drug abuse was scored as the second most important problem in Hawaii at both time points. Hawaii has the 16th lowest rate of mortality from drug overdose in the US with a rate of 10.9/100,000 (16). Concern for pedestrian safety dropped significantly between 2007 and 2013. No significant trends were observed in pedestrian fatality rates between 2008 and 2012. Hawaii has the highest pedestrian fatality rate for seniors in the nation and the fifth highest overall (17). Government responses to natural disasters and pandemic influenza also significantly decreased from 2007. The decrease in concern for pandemic influenza may have been reduced by the fairly mild H1N1 pandemic of 2009. Although an estimated 280,000 people died from the disease, most of these did not occur in the US (18). The Fukushima Daiichi tsunami and nuclear disaster of 2011 do not appear to have strongly influenced the perception of government response to natural disasters.

Obesity was the only public health issue where increased concern was seen across the two time points. Adult obesity in Hawaii was stable over the period (21.7% in 2007 and 21.8% in 2013) and Hawaii has the second lowest obesity rate in the nation (19). This may have been due in part to the efforts of the Hawaii Physical Activity and Nutrition Coalitions. In addition, a state task force on childhood obesity was launched in 2012 and submitted twelve policy recommendations for the 2013 legislative session (20). In addition, First Lady Michelle Obama launched her widely publicized Let’s Move Campaign during this time period.

There were very few differences by type of position or how liberal or conservative individual’s thought were. The sample was almost entirely democratic or non-partisan, but appointed by a democratic governor so variability on these items was somewhat limited. However, most of the correlations were <0.10 indicating little relationship with these variables. We had hypothesized that a change from the Republican governor to a Democratic governor would have influenced the results toward a higher priority for public health issues. This hypothesis was not supported.

Overall, there was little concordance between public health data and the importance of issues. Rating of importance of public health issues was not supported by public health data. It is unclear from this study, what is driving the priorities of decision makers in Hawaii. Further qualitative studies are needed to assess how priorities are formed among decision makers and how they can be affected by advocacy efforts. Public health advocates should consider multi-level approaches that include not only making the data-based case for policy but also the emotional case.

### Limitations

This study had several limitations. Most important is that this was a single group pre- and post-test design. The results show changes in the perceived importance of a variety of issues, however the change may not be due to the economic recession but may be influenced by other non-measured factors. Also, the response rate, especially at time two, may have affected the results, since we are unable to assess the views of the individuals that did not return the survey. In addition, as is true with many surveys of elected officials, the respondent may not have been the officer holder but instead a staff member who is responding for them. Finally, as with any study of elected officials, the results may represent their official positions rather than their personal views.

## Conclusion

This study showed that the relative importance of several public health issues among elected and appointed officials in Hawaii declined between 2007 and 2013. These results indicate that public health advocacy efforts need to be consistent and that attitudes toward public health issues may improve or decline over time. Advocates should also consider the broader context in which policymaking is occurring and seek windows of opportunity when it is advantageous to advance certain policies.

## Author Contributions

JM conceived of the study, conducted the data analysis and led the writing of the paper. MM contributed to the design of the study, led the data collection effort, drafted sections of the manuscript and contributed to the intellectual content of the manuscript. TL participated in the data collection and contributed to the intellectual content of the manuscript. All authors approved the final draft of the manuscript and agree to be accountable for all aspects of the work in ensuring that questions related to the accuracy or integrity of any part of the work are appropriately investigated and resolved.

## Conflict of Interest Statement

The authors declare that the research was conducted in the absence of any commercial or financial relationships that could be construed as a potential conflict of interest.
